# Protein Network Signatures Associated with Exogenous Biofuels Treatments in Cyanobacterium *Synechocystis* sp. PCC 6803

**DOI:** 10.3389/fbioe.2014.00048

**Published:** 2014-11-03

**Authors:** Guangsheng Pei, Lei Chen, Jiangxin Wang, Jianjun Qiao, Weiwen Zhang

**Affiliations:** ^1^Laboratory of Synthetic Microbiology, School of Chemical Engineering and Technology, Tianjin University, Tianjin, China; ^2^Key Laboratory of Systems Bioengineering, Ministry of Education of China, Tianjin, China; ^3^SynBio Research Platform, Collaborative Innovation Center of Chemical Science and Engineering, Tianjin, China

**Keywords:** biofuels, network, WGCNA, tolerance, *Synechocystis*

## Abstract

Although recognized as a promising microbial cell factory for producing biofuels, current productivity in cyanobacterial systems is low. To make the processes economically feasible, one of the hurdles, which need to be overcome is the low tolerance of hosts to toxic biofuels. Meanwhile, little information is available regarding the cellular responses to biofuels stress in cyanobacteria, which makes it challenging for tolerance engineering. Using large proteomic datasets of *Synechocystis* under various biofuels stress and environmental perturbation, a protein co-expression network was first constructed and then combined with the experimentally determined protein–protein interaction network. Proteins with statistically higher topological overlap in the integrated network were identified as common responsive proteins to both biofuels stress and environmental perturbations. In addition, a weighted gene co-expression network analysis was performed to distinguish unique responses to biofuels from those to environmental perturbations and to uncover metabolic modules and proteins uniquely associated with biofuels stress. The results showed that biofuel-specific proteins and modules were enriched in several functional categories, including photosynthesis, carbon fixation, and amino acid metabolism, which may represent potential key signatures for biofuels stress responses in *Synechocystis*. Network-based analysis allowed determination of the responses specifically related to biofuels stress, and the results constituted an important knowledge foundation for tolerance engineering against biofuels in *Synechocystis*.

## Introduction

Human society has been dependent on fossil fuels for centuries. However, fossil fuels are not an infinite resource, and the possibility of their running out in the future and the increasing concerns over energy security and global climate change pose an urgent call for developing renewable ways to produce fuels. Among all alternatives, photosynthetic cyanobacteria have recently attracted significant attention as a promising “microbial cell factory” to produce renewable biofuels due to their capability to utilize solar energy and CO_2_ as the sole energy and carbon sources, respectively (Ducat et al., 2011; Quintana et al., 2011; Robertson et al., 2011). Cyanobacteria contain considerable amounts of lipids in the thylakoid membranes and possess higher photosynthetic efficiency and faster growth rate compared to eukaryotic green algae and higher plants (Quintana et al., 2011). In addition, cyanobacteria have a relatively simple genetic background and are amenable to modification by metabolic engineering and synthetic biology (Wang et al., 2012a). Recent efforts have led to successful production of various biofuels in engineered cyanobacterial cells, such as ethanol (Deng and Coleman, 1999), butanol and isobutanol (Atsumi et al., 2009), alkanes (Choi and Lee, 2013), and biodiesel (Da Ros et al., 2013). However, the current biofuel productivity in the cyanobacterial systems is several orders of magnitude lower than their native producing microbes (Jin et al., 2014). In addition to ongoing efforts to optimize the existing pathways and to discover and construct novel pathways, one option to achieve high productivity is to improve cellular tolerance to toxic biofuel products synthesized by the cyanobacterial hosts (Dunlop, 2011; Zingaro and Papoutsakis, 2012).

Although response mechanisms against biofuels have been extensively studied in many native biofuel-producing microbes (Couto et al., 1997; Dunlop, 2011), it remains unclear for cyanobacteria. As part of our long-term goal to construct more robust and product-tolerant photosynthetic “*chassis*” for synthesizing various renewable biofuels, our laboratory has applied integrated transcriptomic, proteomic, and metabolomic approaches to determine the metabolic profiles of a model cyanobacterium *Synechocystis* sp. PCC 6803 (hereafter *Synechocystis*) stressed under various biofuels (Liu et al., 2012; Qiao et al., 2012; Wang et al., 2012b; Tian et al., 2013; Zhu et al., 2013). Consistent with early genome-level studies in other microbes (Nicolaou et al., 2010; Dunlop, 2011), our previous results showed that *Synechocystis* cells employed a combination of multiple resistance mechanisms in dealing with biofuels stress (Wang et al., 2012b). In addition, the comparative proteomic analysis provided strong evidence that proteins involved in multiple aspects of photosynthesis (i.e., photosystems I and II, cytochrome, and ferredoxin) were up-regulated in ethanol-treated *Synechocystis* (Qiao et al., 2012), suggesting there could be unique response mechanisms employed by cyanobacteria to combat biofuel toxicity.

Although initial efforts using a conventional approach of analyzing individual genes/proteins according to fold change and statistical significance has led to determination of the responses associated with each of the biofuels in *Synechocystis* (Liu et al., 2012; Qiao et al., 2012; Wang et al., 2012b; Tian et al., 2013; Zhu et al., 2013), it becomes clear that network-focused rather than individual gene/protein-focused methodologies would be more appropriate to obtain a complete picture of cellular response (Lehtinen et al., 2013). In addition, the network analysis defines modules and their possible biological roles based on connectivity of proteins or genes rather than using any artificial cutoff, which may avoid information loss related to genes/proteins of low abundance or small fold changes, such as signal transduction genes. In recent years, network analysis has been applied to cyanobacterial studies. For example, Singh et al. (2010) constructed a *Bayesian* network of *Synechocystis* using transcriptomic data and defined a set of genes as the core transcriptional response (CTR) that are commonly regulated under most of environmental perturbations (Singh et al., 2010). McDermott et al. (2011) developed a predictive *in silico* model of diurnal and circadian behavior of *Cyanothece* 51142 using transcriptomic data, and the results showed that incorporation of network topology into the model could improve the ability to explain the behavior (McDermott et al., 2011). Recently, Wang et al. (2013b) utilized a weighted gene co-expression network analysis (WGCNA) approach to establish transcriptional networks for four cyanobacterial species under metal stresses, and a further cross-species network comparison led to the discovery of several core response modules and genes that may be essential to all metal stresses, as well as species-specific hub genes for metal stresses (Wang et al., 2013b). The studies demonstrated that network-based analysis could be a powerful tool in deciphering cellular responses.

In this study, to further identify responses specifically related to biofuels stress that could be used as potential targets for rational tolerance engineering, a topological analysis of global proteins co-expression network combined with protein–protein interaction (PPI) network was first performed to uncover a core set of proteins commonly responsive to both biofuels stress and environmental perturbations. Then, a WGCNA was applied to identify responses specifically related to biofuels stress. The combination of both analyses allowed the identification of the protein network signatures associated with exogenous biofuels treatments, and provided new insights into the molecular mechanisms against biofuels stress in *Synechocystis*.

## Materials and Methods

### Proteomic data sources

A total of five iTRAQ LC-MS/MS datasets of *Synechocystis* sp. PCC 6803 from our previous study were re-analyzed at a peptide level. Growth of *Synechocystis* under ethanol, butanol, hexane, salt stress conditions with dosages of 1.5% (*v*/*v*), 0.2% (*v*/*v*), 0.8% (*v*/*v*), 4% (*w*/*v*), and nitrogen starvation, which led to ~50% growth reduction were then determined. For each condition, cells were harvested at two time points (24 and 48 h) that were corresponding to middle-exponential and exponential-stationary transition phases in the growth time courses for proteomics analysis. Each biological replicates sample has two technical replicates. Due to the page limitation, for details about the environmental perturbation and biofuel stress experiments and original proteomic datasets please find from several previous publications (Liu et al., 2012; Qiao et al., 2012, 2013; Huang et al., 2013; Tian et al., 2013).

### Proteomic data analysis

The mass spectroscopy analysis was performed using a AB SCIEX TripleTOF™ 5600 mass spectrometer (AB SCIEX, Framingham, MA, USA), coupled with online micro flow HPLC system (Shimadzu Co, Kyoto, Japan) as described previously. Genome sequence and annotation information of *Synechocystis* sp. PCC 6803 were downloaded from NCBI (ftp://ftp.ncbi.nlm.nih.gov/genomes). The details for the experimental design, execution, and proteomic data analysis can be found in the original publications (Liu et al., 2012; Qiao et al., 2012, 2013; Huang et al., 2013; Tian et al., 2013).

### Protein co-expression network construction

To construct the association network from proteomic data, we used a multi-step procedure for network construction: first, we performed a procedure for data normalization identical with Principal component analysis (PCA) (See below); second, correlation values were calculated between present values for all pairs of peptides. In this study, we used peptides rather than proteins to construct the protein co-expression network. One reason is lots of related peptides from the same protein are always observed in discordance, which may be due to different post-translational modifications or isoforms. Correlation is calculated as the *Pearson* correlation coefficient for all pairwise peptides. Third, in order to generate a reliable protein co-expression network, high correlation coefficients (*r* > 0.9) was used, where only gene pairs with a correlation coefficient higher than 0.9 were considered connected. Finally, we combined protein co-expression network with experimentally determined PPI network (Sato et al., 2007). In this process, known PPI between observed proteins already in the co-expression network were added as new edges to the network.

### Topological analysis

Topological analysis of networks was performed using Cytoscape software. Bottleneck and hub proteins were defined as the top 20% of proteins ranked by the values of betweenness and degree centrality, respectively (McDermott et al., 2011, 2012). The degree and betweenness centrality metrics were defined according to the methods described by McDermott et al. (2012). Briefly, degree centrality is a metric of the connectedness of a node, and betweenness centrality is a metric that measures how often paths between nodes must traverse a given node. Generally, degree centrality is the fraction of edges for a particular protein out of all possible interactions for that protein in the network, and betweenness is the number of shortest paths between all pairs of proteins in the network that pass through a specific node.

### Principal components analysis

The proteomics data were converted to a ratio versus control conditions. The data were then log2 scaled, and each unit reflects a twofold change in abundance. In order to avoid influence caused by missing data, peptides with any missing data in any condition were removed. Remaining core peptides identified in all conditions were subjected to PCA and partial least square-discriminant analysis (PLS-DA) by SIMCA-P 11.5 software. Averaging was taken for all technical replicates of samples, as in general good reproducibility was observed between replicates (Liu et al., 2012; Qiao et al., 2012; Tian et al., 2013).

### Weighted gene co-expression network analysis

Weighted gene co-expression network analysis approach was used to establish a co-expression network from the LC-MS/MS proteomic data (Langfelder and Horvath, 2008). The co-expression network was created first by calculating weighted *Pearson* correlation matrices corresponding to peptide abundance expression, and then by following the standard procedure of WGCNA to create the networks. Briefly, weighted correlation matrices were transformed into matrices of connection strengths using a power function. These connection strengths were then used to calculate topological overlap (TO) (Langfelder and Horvath, 2008). The topological overlap matrix (TOM) is computed as TOM*_ij_* = (*l_ij_* + *a_ij_*)/[min (*k_i_*,*k_j_*) + 1 − *a_ij_*] where *l_ij_* is defined as the dot product on row *i* and column *j* in adjacency matrix [*a*] and *k_i_* (the connectivity) is the summation of row *i* in adjacency matrix [*a*] (Gibbs et al., 2013). Hierarchical clustering based on TO was used to group proteins with highly similar co-expression relationships into modules. Protein dendrograms were obtained by average linkage hierarchical clustering, while the color row underneath the dendrogram showed the module assignment determined by the Dynamic Tree Cut method. The network for each module was generated with the minimum spanning tree with a dissimilarity matrix from WGCNA. The modules with *r* > 0.55 and a *p*-value <0.1 were extracted (Wang et al., 2013a).

### Functional enrichment analysis

Metabolic pathway enrichment analysis was conducted according to Kyoto Encyclopedia of Genes and Genomes (KEGG) and Cluster of Orthologous Groups of proteins (COG) database using the following formula:

$$p=1-\sum_{i=0}^{m-1}\frac{\left(\begin{array}{c} M\\ i \end{array}\right)\left(\begin{array}{c} N-M\\ n-i \end{array}\right)}{\left(\begin{array}{c} N\\ n \end{array}\right)}$$

*N* is the number of all proteins with KEGG pathway annotation information, *M* is the number of proteins with a given KEGG pathway annotation, *n* is the number of the associated proteins with KEGG pathway annotation information, and *m* is the number of the associated proteins with a given KEGG pathway annotation. All pathway mapping was manually checked for each of the proteins. We also calculated functional enrichment by considering each group or module of interest versus all proteins in the network as a background, as the ratio of *m*/*n* versus *M*/*N*.

## Results and Discussion

### Overview of proteomics analysis

The proteomic datasets used in this study are listed in Table 1. Briefly, the datasets contain four sets of quantitative iTRAQ–LC-MS analyses of *Synechocystis* grown under five stress conditions, i.e., biofuel stresses of ethanol, butanol and hexane, and environmental perturbations of high salt and nitrogen starvation. For each condition, treated and corresponding wild-type control cells were harvested at two time points (i.e., 24 and 48 h). Each biofuel-stressed dataset has two technical replicates. For overall data quality, reproducibility and full description of the proteomic datasets, please refer to several previous publications (Liu et al., 2012; Qiao et al., 2012, 2013; Huang et al., 2013; Tian et al., 2013).

**Table 1:** **The proteomic datasets used in this study**.

Condition^a^	Unique spectra	Peptides	Unique peptides	Proteins
Ethanol (Qiao et al., 2012)	21,066	7,337	7,192	1,523
Butanol (Tian et al., 2013)	18,745	6,355	6,252	1,300
Hexane (Liu et al., 2012)	19,217	6,995	6,875	1,389
Salt (Qiao et al., 2013)	23,822	8,379	8,257	1,702
N-starvation (Huang et al., 2013)	23,674	8,404	8,282	1,703

*^a^References for each dataset are provided*.

In previous studies, all identified peptides were matched to proteins in the *Synechocystis* genome, and then further analysis was conducted using protein-based quantitative data (Liu et al., 2012; Qiao et al., 2012, 2013; Huang et al., 2013; Tian et al., 2013). However, recent studies showed that the peptide-based proteomic data can be a better choice in constructing protein network since peptides derived from the same protein were shown to have a statistically higher TO and concordance in abundance, which is potentially important for inferring protein abundance (Gibbs et al., 2013). In addition, using peptide-based data also avoids issues related to multiple mapping of the same peptide (Cox and Mann, 2011; Gibbs et al., 2013). In this study, we thus used the peptide-based raw proteomic data and subjected them directly to Mascot analysis. After data filtering to eliminate low-scoring spectra, only the peptides that were identified in both control and the stress-treated samples (so that the ratio calculation is possible) were included for further analysis, resulting a final dataset consisting of 11,179 unique peptides, which are corresponding to 1,971 proteins.

**Figure 1:**
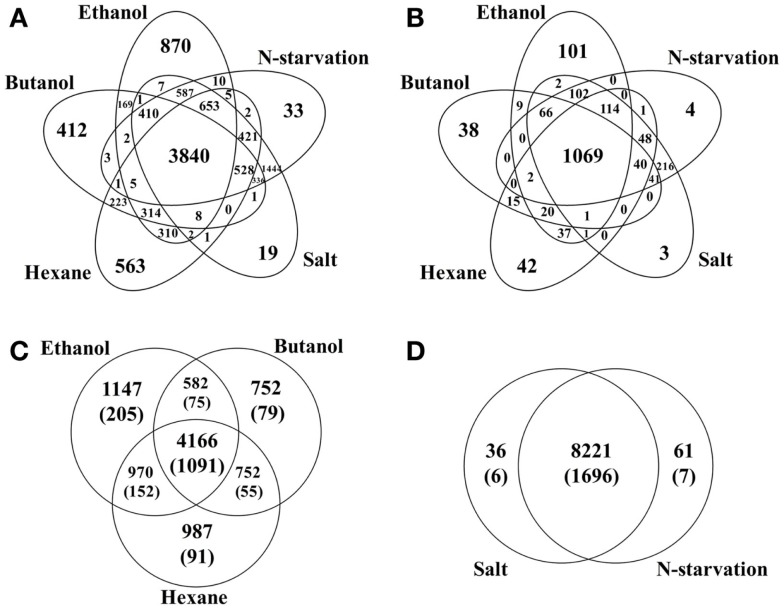
**Comparisons of peptides identified under different stress conditions**. **(A)** Peptides distribution among conditions, including ethanol-, butanol-, hexane-, high salt, and nitrogen starvation treated samples. **(B)** Proteins distribution among conditions. **(C)** Peptides/proteins distribution among biofuels stress conditions. Protein-based information was provided inside parenthesis. **(D)** Peptides/proteins distribution between two environmental perturbations. Protein-based information was provided inside parenthesis.

Comparison between various stress conditions showed that a total of 3,840 peptides that correspond to 900 (22.7%) proteins were identified in all conditions, possible core stress responses in *Synechocystis*. Functional classification of these commonly identified proteins showed that they were found in almost all aspects of *Synechocystis* metabolism (Additional File S1 in Supplementary Material). Comparison of these possible core stress response proteins with the CTR identified previously (Singh et al., 2010) showed that 230 of the 399 CTR proteins were also responsive in all stress conditions of this study. In addition, the comparison allowed identification of the proteins associated with each individual or multiple stress conditions (Figure 1). For example, ethanol-, butanol-, and hexane-stressed datasets shared a common set of 4,166 peptides, corresponding to 1,091 proteins, while each contained 1,474, 752, and 987 unique peptides, respectively; the environmental perturbations of high salt and nitrogen starvation shared a common set of 8,221 peptides, while each contained only 36 and 61 unique peptides, respectively. The great difference in terms of the number of unique peptides between biofuels stress and environmental perturbation suggested that different response strategies could be employed in *Synechocystis* (Singh et al., 2010).

### Construction of protein co-expression network to identify common responses

**Figure 2:**
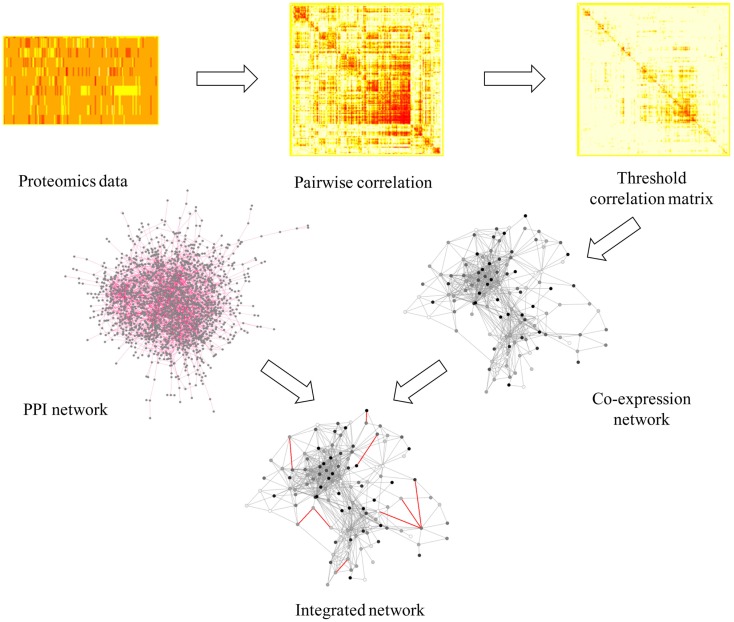
**A scheme of workflow to generate protein co-expression network**. In integrated network, gray line represents co-expression information and red line represents PPI information.

The network approach combined with topological analysis of global “omics” datasets has been proven to be a significant tool to identify responses under multiple different conditions (McDermott et al., 2012), in many organisms such as cyanobacteria, pathogenic bacteria, yeast, worm, fly, and human cell culture (McDermott et al., 2012). Previous analyses of stress responses to exogenous biofuels in *E. coli* (Dunlop, 2011; Wang et al., 2013a; Jin et al., 2014) and cyanobacteria (Liu et al., 2012; Qiao et al., 2012; Tian et al., 2013) using conventional methodologies showed that both general stress responses such as up-regulation of heat shock proteins and membrane modification, and possible biofuel-specific responses can be induced by individual biofuel stress. To further decipher metabolic responses using a network-based approach, we first constructed a protein co-expression network using proteomic datasets to determine the general stress responses that were commonly responsive to both biofuels stress and environmental perturbations. Briefly, the protein co-expression network was constructed based directly on pairwise or low-order conditional pairwise association measures, such as the correlation or mutual information, to infer the connectivity between proteins (Nicolaou et al., 2010). This method has the advantage of low computational complexity, which is a more suitable approach for network analysis of relatively large number of quantitative peptide data in this study (Nicolaou et al., 2010). The workflow of the network construction was illustrated in Figure 2. First, we inferred the network using the similarities between expression profiles of all qualified peptides through *Pearson* correlation and then filtered the correlations to remove those with low correlation values. Here, we used a relatively high threshold of 0.9 to ensure a highly credible connection between peptides, while avoiding losing too many nodes. Second, we transformed the threshold correlation matrix into a peptide co-expression network using a *perl* script (available upon request). The nodes in the networks are peptides while the links between them (edges) represent co-expression properties. The result showed that most of the peptides from the same protein tended to cluster together, which can be viewed as a validation of the network quality. In several cases, we observed that peptides from the same proteins were located in discordance in the network, which is probably due to the different post-translational modifications to the same proteins (Gibbs et al., 2013). Third, as a previous study showed that incorporating a PPI network into a proteins co-abundance network could significantly improve target discrimination using topological measures than the networks without PPI (McDermott et al., 2012), we further integrated a PPI network of *Synechocystis* constructed by Sato et al. (2007) to the protein co-expression network we constructed by adding them directly as new edges in the protein co-expression network. The integrated network achieved has a total of 866 nodes and 20,226 edges, representing a majority of the proteins (866/900) we identified from all stress conditions.

Topological analysis was then conducted by calculating topological attributes of all the nodes in the network. It is well accepted that nodes with top degrees and betweenness are highly central in networks, and so-called hubs and bottlenecks are more likely to be important to the system than others with low topological attributes (Sato et al., 2007; Yao and Rzhetsky, 2008). Based on the same criteria used in several previous studies (McDermott et al., 2011, 2012), we determined bottlenecks as the proteins in the network with top 20% of the betweenness values and hubs as the proteins with top 20% of the degrees values. Interestingly, the result showed that most proteins (109/180) were with both top 20% betweenness and top 20% degrees, thus considered to be bottleneck-hubs (Sato et al., 2007), consistent with a previous study that showed high correlation between betweenness of a node with its corresponding degrees (Goh et al., 2003).

### Functional characterization of bottleneck and hub proteins

**Figure 3:**
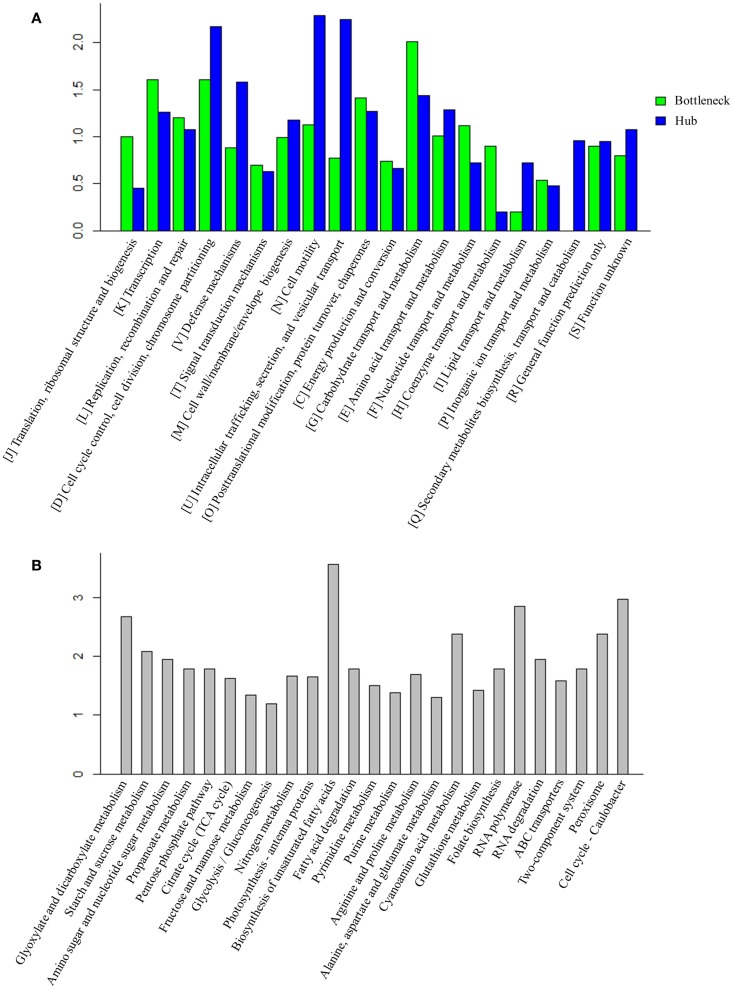
**Function analysis of bottlenecks and hubs proteins**. **(A)** COG enrichment analysis of bottlenecks and hubs proteins. *X*-axis indicates the COG category and *Y* -axis indicates the ratio compared to background. **(B)** KEGG metabolic pathways enrichment analysis of bottlenecks and hubs proteins. Top 25 metabolic pathways are listed and ordered by function category. *X*-axis indicates the names of pathway and *Y* -axis indicates the ratio compared to background.

Early studies have found that common stress responses typically involve wide aspects of cell metabolism, including induction of oxidative stress response, heat shock proteins, efflux pumps, and accumulation of osmoprotective compounds (Nicolaou et al., 2010; Rutherford et al., 2010; Dunlop, 2011). As the hubs and bottlenecks identified from the integrated protein network are highly relevant to the stress responses, we conducted an enrichment analysis of bottlenecks and hubs among functional categories (Figure 3A). The results showed that both bottlenecks and hubs had a very similar pattern of being highly associated with several key functional categories, such as “[K] Transcription,” “[L] Replication, recombination and repair,” and “[O] Post-translational modification, protein turnover, chaperones.” Meanwhile, the results also showed that the bottlenecks were highly associated with “[G] Carbohydrate transport and metabolism” functional category, while the hubs were highly associated with “[D] Cell cycle control, cell division, chromosome partitioning,” “[N] Cell motility, ” and “[U] Intracellular trafficking, secretion, and vesicular transport” functional categories.

Enrichments of the bottlenecks and hubs among several metabolic pathways were also observed (Figure 3B**)**. Briefly, the enrichments are described below: (i) *ROS response pathway*: early studies showed organic solvent or environment stress induced production of reactive oxygen species (ROS) in many microbes (Houot et al., 2007; Stanley et al., 2010; Yang et al., 2010; Wang et al., 2013b). ROS accumulation could lead to DNA mutation, mRNA and protein denaturation, and membrane lipid peroxidation and ultimately cell death (Bhattacharya et al., 2004). Generation of antioxidants, such as superoxide dismutase (SOD) (Bhattacharya et al., 2004), glutaredoxin (Marteyn et al., 2013), carotenoids (Wilson et al., 2006), or tocopherols (Yang et al., 2008) that were capable of rapidly detoxifying ROS, has been considered as one of the key strategies to deal with stress in *Synechocystis* (Bhattacharya et al., 2004; Wilson et al., 2006; Yang et al., 2008; Marteyn et al., 2013). The results showed that the pathways related to antioxidants response, such as “Peroxisome” (ko04146) and “Glutathione metabolism” (ko00480), were enriched in higher TO proteins, consistent with the recent discovery that peroxiredoxins and glutathione-dependent peroxidase play major roles in combating oxidative stress in cyanobacterium *Anabaena* (Banerjee et al., 2012). Peroxisomes could convert hydrogen peroxide to water and thus protect the microorganism from oxidative damage, while glutaredoxin could catalyze the reduction of protein disulfides and glutathione-protein mixed disulfides in a coupled system with glutathione, NADPH, and glutathione reductase (Li et al., 2005); (ii) *Transporters*: transporters have been suggested as one important mechanism against solvent/biofuel toxicity in *Synechocystis* (Tian et al., 2013). For example, *slr1295* encoding an iron transport system substrate-binding protein was involved in butanol resistance (Zhu et al., 2013). In addition, a broad range of transporters with different substrate-specificities were also found involved in organic solvent tolerance in *E. coli* (Okochi et al., 2007). Moreover, transporters were also involved in tolerance to many environmental perturbations. For example, *ggtA* gene (*slr0747*) encodes a subunit of the transport system for the osmoprotective compound glucosylglycerol that is necessary for *Synechocystis* grown under salt stress (Hagemann et al., 1997). Our analysis found that “ABC transporters” (ko02010) was enriched in hubs and bottlenecks, suggesting that transporters play an essential role for cell survival when grown under a wide range of stresses; (iii) *Cell membrane permeability*: as a common resistance barrier against environmental stresses, changes of cell wall, or cell membrane composition can improve solvent tolerance to biofuels in many microbes (Ramos et al., 1997; Kajiwara et al., 2000; Zhao et al., 2003). In addition, an early study showed that unsaturation of fatty acids was associated with the ability of the photosynthetic machinery to tolerate salt stress in *Synechocystis* (Allakhverdiev et al., 1999). Consistent with this result, the network analysis also found that pathways “Biosynthesis of unsaturated fatty acids” (ko01040) and “Fatty acid degradation” (ko00071) were enriched in the bottlenecks and hubs.

### WGCNA analysis to determine the biofuel-specific responses

**Figure 4:**
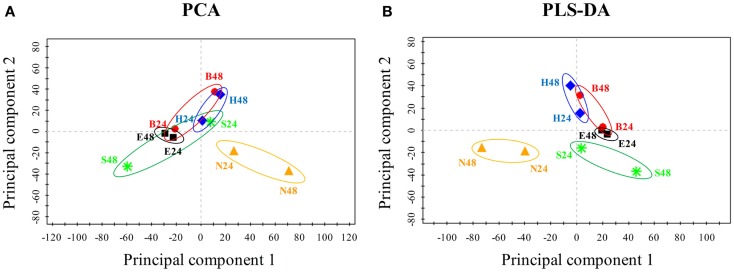
**Principal component analysis and PLS-DA score plot of the responses under various stress conditions in *Synechocystis***. **(A)** PCA score plot. Samples with different treatments were indicated by colors. *X*-axis, *Y* -axis were labeled with the first principal component and the second principal component, accounting for 33.4 and 16.3% total variation, respectively. **(B)** PLS-DA score plot.

To uncover biofuel-specific responses, we first converted the raw proteomic data into ratio data between the stress and the control conditions, and then used the log2 transformed ratio datasets for a PCA analysis. PCA score plot showed that almost all samples (i.e., different treatments, time points) were visibly separated, suggesting there are obvious differences in terms of the metabolic responses between various biofuels stress and environmental perturbations (Figure 4A). In addition, PCA score plot also revealed: (i) samples at 24 h of the middle-exponential phase tended to be clustered together, while samples at 48 h of the exponential-stationary transition phase were distinctly separated and became far away from the center when compared to those of 24 h, suggesting that more dramatic metabolic changes occurred after stress treatments of longer time; (ii) a greater separation along principal component 2nd between the biofuel-treated and environmental-treated samples was observed, and the biofuels-stressed profiles tended to be clustered together when compared to salt and nitrate starvation, suggesting there is a relatively high similarity between all biofuels-stressed samples than to environmental perturbations; (iii) a moving trend of the profiles along the principal component 1st seemed correlated with the carbon chain length of the biofuels tested in this study, although further proof is still needed; and (iv) finally, it was also observed that one of the salt treatment samples was clustered closely with biofuel-stressed samples, suggesting that just PCA analysis itself may not be enough to determine the biofuel-specific responses. Subsequently, we also performed a PLS-DA to further define differences between responses to various stresses. In the PLS-DA score plot, all biofuel samples are more tightly clustered together, completely separated with samples perturbed by environmental stresses (Figure 4B), indicating clear differences between the sample groups of biofuel and environmental stresses and suggestive of the different metabolic responses.

**Figure 5:**
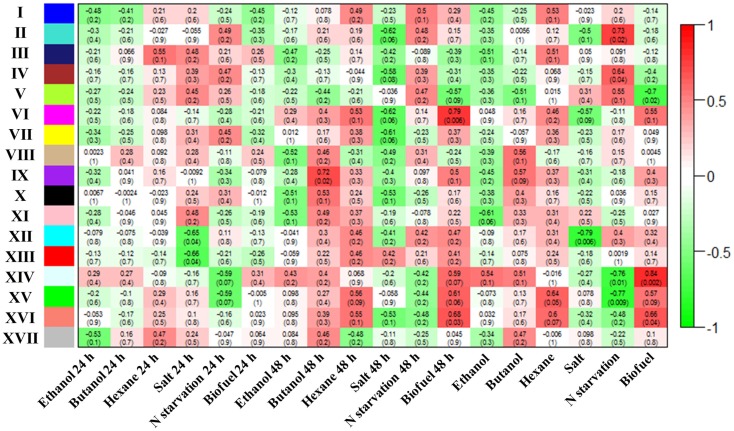
**Correlation between identified modules and stress conditions**. Correlation of each module with conditions was indicated by colors. In addition, correlation efficient of modules with conditions are provided for each module, and the *p*-values are provided inside parenthesis.

Weighted gene co-expression network analysis was employed as a method that can additionally define “modules” of co-expressed proteins explicitly and provide additional network statistics that describe the systems properties of metabolic networks (Langfelder and Horvath, 2008). The WGCNA analysis showed that a total of 17 metabolic modules were detected within the WGCNA co-expression networks of *Synechocystis*. Using a cut-off of correlation coefficients (*r* value > 0.55) and their confidence (*p*-values < 0.1), we found 5 out of 17 modules correlated with biofuels stress, among which 4 module’s eigengenes were positively correlated while only 1 module eigengenes were negatively correlated with biofuels stress (Figure 5**)**. A scatter plot of peptide significance versus module membership was plotted for these biofuel related modules (Additional Figure S1 in Supplementary Material), and the results also demonstrated high correlations between biofuel and the respective module eigengenes. In contrast, the background peptides (module XVII) showed no correlation with any biofuel stress. Among all modules positively correlated to biofuels stress, module VI eigengenes were negatively correlated with salt stress; module XIV and XV eigengenes were negatively correlated with nitrate starvation; and module XVI eigengenes were negatively correlated with both salt and nitrate starvation stress.

Topological overlap is a similarity metric that incorporates information from neighboring nodes, making it robust to noisy correlations. TOM is a robust and biologically meaningful measurement that encapsulates the similarity of a co-expression relationship with all others in the network (Langfelder and Horvath, 2008). Within the network constructed by the WGCNA approach, we first trimmed the network with a moderate threshold, removing low-quality connections with strengths between nodes less than TO value of 0.1. The results showed that, even after the threshold removal, most of the connections were still kept in the network; for example, the XV module was only reduced to 250 nodes from the initial 264 nodes (data not shown). Topological analysis was then conducted to determine the top 10 hub and bottleneck peptides for each biofuel-correlated modules. The result showed most peptides with higher topological attributes were related to proteins of photosynthesis functions in the biofuel-correlated modules (data not shown).

**Figure 6:**
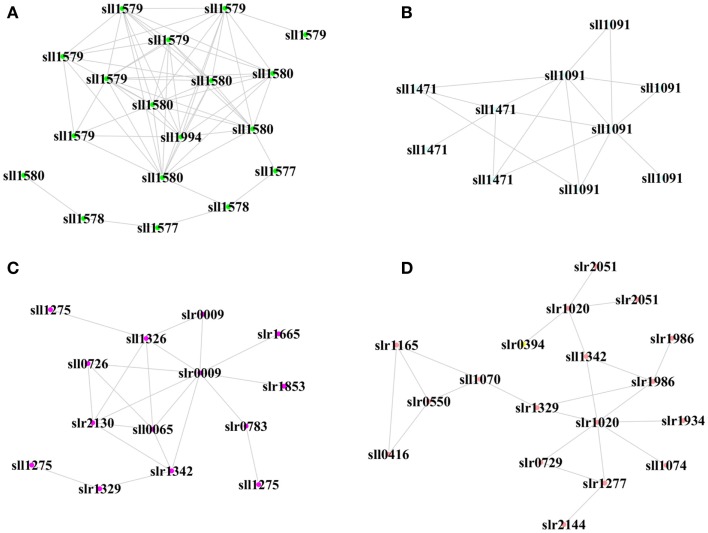
**Hub proteins and their neighbor proteins in biofuel-responsive modules**. **(A)** Hub peptide from Sll1580 “phycocyanin associated linker protein” in the XV module. **(B)** Hub peptide from Sll1091 “43 kD bacteriochlorophyll synthase subunit” in the XIV module. **(C)** Hub peptide from Slr0009 “ribulose-bisphosphate carboxylase” in the VI module. **(D)** Hub peptide from Slr1020 “sulfolipid biosynthesis protein SqdB” in the XVI module. Each node represents a peptide from a protein.

We also determined the top one or two hub peptides with the greatest connectivity in each module, which were supposed to be biologically important under a more stringent threshold (Figure 6). In the XV module positively correlated with biofuels stress, a hub peptide “AITTAASR” from *sll1580* encoding “phycocyanin associated linker protein” was connected with peptides from proteins of the same operon, including *sll1577* “phycocyanin subunit B,” *sll1578* “phycocyanin a subunit,” and *sll1579* “phycocyanin associated linker protein.” In the XIV module positively correlated with biofuels stress, two hub peptides, “TVVPANPLVQMK” and “VALVGDAAGTVTK” were identified. They were matched to the same Sll1091 protein of “a bacteriochlorophyll synthase subunit” and connected with a peptide from *sll1471* encoding “phycobilisome rod-core linker polypeptide.” Together, the results suggested that light-harvesting proteins could be key components involved in biofuels stress response. Interestingly, one peptide “LTYYTPDYTPK” from Slr0009 of “ribulose bisphosphate carboxylase” that catalyzes the first reaction of CO_2_ fixation was also identified as a hub peptide in the VI module positively correlated with biofuels stress. In addition, a hub peptide “VFNQYTELFSVGDLAQMVQK” of Slr1020 in the XVI module positively correlated with biofuels stress was connected to several peptides of proteins related to carbon fixation, such as Sll1342, Slr0394, and Sll1070. The finding of proteins related to CO_2_ fixation function as important hub peptides under biofuels stress implicated that CO_2_ metabolism could also be important in dealing with biofuels stress in cyanobacteria.

### Pathway enrichment analysis of biofuel-specific modules

**Figure 7:**
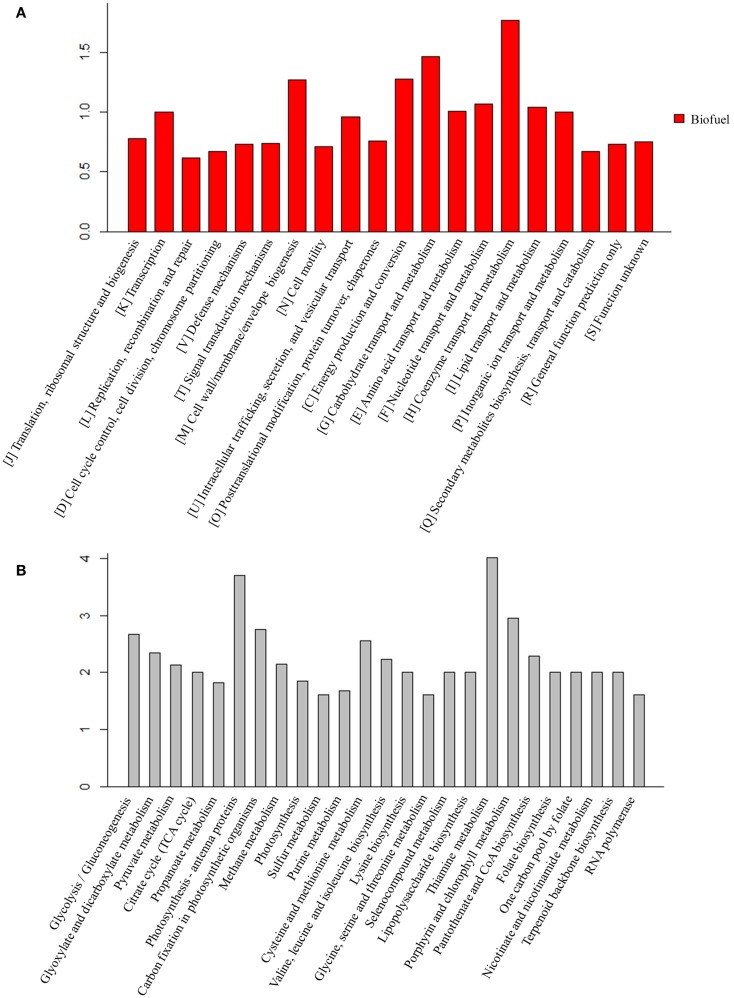
**Enrichment analysis of the proteins identified in biofuel-responsive modules**. **(A)** COG enrichment analysis of proteins in biofuel-responsive modules. *X*-axis indicates the COG category and *Y* -axis indicates the ratio compared to background. **(B)** KEGG metabolic pathways enrichment analysis of proteins in biofuel modules. Top 25 metabolic pathways are listed and ordered by function category. *X*-axis indicates the names of pathway and *Y* -axis indicates the ratio compared to background.

For the peptides/proteins located in the biofuel-associated modules, we carried out an enrichment analysis for their distribution among functional categories and metabolic pathways. The detailed enrichment information for all COG and top 25 KEGG pathways is provided in Additional Files S3 and S4 in Supplementary Material. The results showed that several functional categories were significantly enriched, such as functional categories of “[I] Coenzyme transport and metabolism,” “[G] Carbohydrate transport and metabolism,” “[C] Energy production and conversion,” and “[M] Cell wall/membrane/envelope biogenesis” (Figure 7A).

The KEGG pathway enrichment analysis showed that “Lipopolysaccharide biosynthesis” (ko00540), “Photosynthesis – antenna proteins” (ko00196), “Porphyrin and chlorophyll metabolism” (ko00860), and “Carbon fixation in photosynthetic organisms” (ko00710) were significantly enriched (Figure 7B). Structures and components of the cell wall are known to be affected by solvents/biofuels (Nicolaou et al., 2010; Dunlop, 2011; Jin et al., 2014). Early analysis of cell morphology found visible cell aggregation under biofuel treatments (Liu et al., 2012; Qiao et al., 2012; Tian et al., 2013), while the aggregation was not obvious under environmental stress (Huang et al., 2013; Qiao et al., 2013). Consistently, an early study found that the mutants of *E. coli* with hyper tolerance under organic solvent stress tend to increase lipopolysaccharide content (Aono and Kobayashi, 1997). Phycobilisomes and phycocyanins are the most abundant proteins and the major light-harvesting antennae for photosynthesis in cyanobacteria (Nakamoto and Honma, 2006) and are directly attached to the thylakoid membranes, where they absorb photons and efficiently transfer excitation energy to the photosynthetic reaction centers (Nakamoto and Honma, 2006). Twelve of 13 identified proteins in pathway “Photosynthesis – antenna proteins” (ko00196), and 17 of 37 proteins in pathway “Photosynthesis” (ko00195) were up-regulated, respectively. In addition, two other photosynthesis related pathways, “Porphyrin and chlorophyll metabolism” (ko00860) and “Terpenoid backbone biosynthesis” (ko00900) that is a precursor biosynthetic pathway of accessory pigment-carotenoid, were also up-regulated specifically in response to biofuels. In early studies, carotenoid biosynthesis has been found up-regulated by strong light stress in *Synechococcus elongatus* PCC 7942 (Schafer et al., 2006) and in stress-tolerant mutants of *Haematococcus pluvialis* (Sandesh Kamath et al., 2008). Moreover, Slr1225 protein of phytoene synthase involved in carotenoid biosynthesis was also identified in the V module positively correlated to biofuels stress. Similarly, 11 of 16 proteins in “Carbon fixation in photosynthetic organisms” (ko00710) pathway were also up-regulated by the biofuel. These results were consistent with early studies that showed dissociation of phycobilins from the thylakoids in *Anabaena* P-9 under external free fatty acids biotic stress (Wu et al., 2006) and *S. elongatus* PCC 7942 (Ruffing and Jones, 2012), and the proteins associated with photosynthesis, especial PSII, up-regulated during free fatty acids production in *S. elongatus* PCC 7942 (Ruffing, 2013).

The KEGG pathway enrichment showed amino acid metabolism related pathways, including “Glycine, serine, and threonine metabolism” (ko00260), “Cysteine and methionine metabolism” (ko00270), “Lysine biosynthesis” (ko00300), and “Valine, leucine, and isoleucine biosynthesis” (ko00290) were significantly enriched (Figure 7B). The roles of amino acid in stress resistance was previously reported in *E. coli*, in which lysine, tryptophan, leucine, isoleucine, and valine were found related to resistance to acid and various biofuel products (Diez-Gonzalez and Karaibrahimoglu, 2004; Horinouchi et al., 2010; Wang et al., 2014), and the addition of isoleucine can improve ethanol tolerance of *E. coli* (Horinouchi et al., 2010). It may be worth to determine whether similar roles are also played by amino acids in cyanobacteria.

The KEGG pathway enrichment showed carbohydrate metabolism related pathways, such as “Pyruvate metabolism” (ko00620), “Glycolysis” (ko00100), “Citrate cycle” (ko00020), and related cofactors metabolism pathway “Thiamine metabolism” (ko00730) were also enriched (Figure 7B). Enrichment of “Glycolysis” (ko00100) was probably due to the fact that most of its proteins are the same as “Carbon fixation in photosynthetic organisms” (ko00710). In addition, although proteins functioning in “Pyruvate metabolism” were up-regulated under biofuels stress, no enzyme after acetyl-CoA in the citrate cycle was found in the biofuel-responsive modules, implying acetyl-CoA was probably more directed into fatty acid biosynthesis rather than the citrate cycle under biofuel stress conditions.

## Conclusion

**Figure 8:**
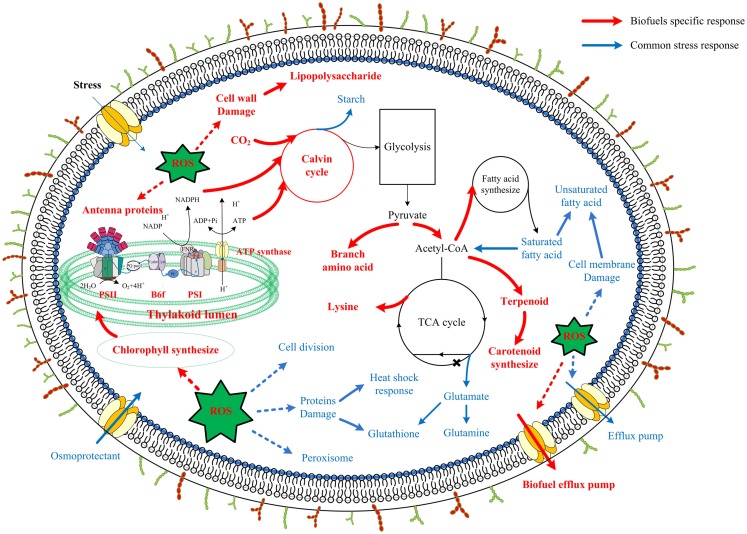
**Scheme of biofuel-responsive signatures in *Synechocystis***. The metabolic features presented were deduced either from proteins integrated network as common stress responses (blue) or from WGCNA analysis as biofuel-specific responses (red). Processes associated with photosynthesis, central carbohydrate metabolism, lipolysis and fatty acid metabolism, amino acid metabolism, ROS stress responses, as well as cell surface structure modification are indicated and labeled. Relevant transport systems are shown within the cell membrane as well. *According to a recent study in *Synechococcus*, function of 2-oxoglutarate dehydroase in citrate cycle was finished by Sll1981 protein of 2-oxoglutarate decarboxylase and Slr0370 protein of a succinic semialdehyde dehydrogenase (Zhang and Bryant, 2011; Xiong et al., 2014).

Although synthetic biology technologies have improved biofuel production significantly in photosynthetic cyanobacteria, current biofuels productivity in these renewable systems is still very low (Oliver and Atsumi, 2014). Meanwhile, it becomes clear that toxicity of the end-product biofuels to cyanobacterial cells may represent a major hurdle for further improving the efficiency and productivity of the processes. For rational construction of high-tolerant *chassis* (Alper et al., 2006), the knowledge on molecular mechanisms responsive to biofuels stress is necessary (Baer et al., 1987; Atsumi et al., 2010). To seek a better understanding of the biofuel-tolerance mechanisms, in this study, using the proteomic datasets collected from several previous studies (Liu et al., 2012; Qiao et al., 2012, 2013; Huang et al., 2013; Tian et al., 2013), we applied network-based methodologies to compare the stress responses induced by three biofuels stress and two environmental perturbations in *Synechocystis*, as the network-based strategy has the advantages of identifying low abundance or small changes and stress-specific response proteins (Singh et al., 2010; McDermott et al., 2011; Wang et al., 2013b). The comparison allowed identification of a set of common responsive proteins to all perturbations, many of which were identical to the core transcriptional genes determined previously in *Synechocystis* (Singh et al., 2010). In addition, the analysis revealed proteins related to cell surface lipopolysaccharide modification, photosynthesis (i.e., photosynthetic pigments, light-harvesting, and carbon fixation), and branch amino acid biosynthesis could be specific responses to biofuels. A scheme of metabolic signatures associated with exogenous biofuels treatments in *Synechocystis* was presented in Figure 8. Briefly, the biofuel-responsive signatures of *Synechocystis* may include enhanced activities associated with transporters, photosynthesis, CO_2_ fixation, ROS detoxification proteins, and some amino acid and acetyl-CoA biosynthesis for fatty acid. The study provided a better view of metabolic responses caused by the biofuels stress, and also demonstrated that the network-based approach is a powerful tool to identify important target proteins responsive to biofuels stress.

## Author Contributions

Guangsheng Pei, Lei Chen, Jiangxin Wang, Jianjun Qiao, and Weiwen Zhang conceived of the study. Guangsheng Pei carried out the data analysis. Guangsheng Pei and Weiwen Zhang drafted the manuscript. All authors read and approved the final manuscript.

## Conflict of Interest Statement

The authors declare that the research was conducted in the absence of any commercial or financial relationships that could be construed as a potential conflict of interest.

## Supplementary Material

The Supplementary Material for this article can be found online at http://www.frontiersin.org/Journal/10.3389/fbioe.2014.00048/abstract

## Abbreviations

COG, cluster of orthologous groups of proteins; CTR, core transcriptional response; HPLC, high performance liquid chromatography; iTRAQ, isobaric tag for relative and absolute quantitation; KEGG, Kyoto encyclopedia of genes and genomes; LC-MS/MS, liquid chromatography-tandem mass spectrometry; PCA, principal component analysis; PLS-DA, partial least square-discriminant analysis; PPI, protein–protein interaction; ROS, reactive oxygen species; SOD, superoxide dismutase; TO, topological overlap; TOM, topological overlap matrix; WGCNA, weighted gene co-expression network analysis.
