# Supplementary material for: Protein Network Signatures Associated with Exogenous Biofuels Treatments in Cyanobacterium Synechocystis sp. PCC 6803
Source: Front Bioeng Biotechnol. 2014 Nov 3;2:48. doi: 10.3389/fbioe.2014.00048 (PMC4217553; doi:10.3389/fbioe.2014.00048)
Supplement: Supplementary file 5 [file Image_1.PDF]

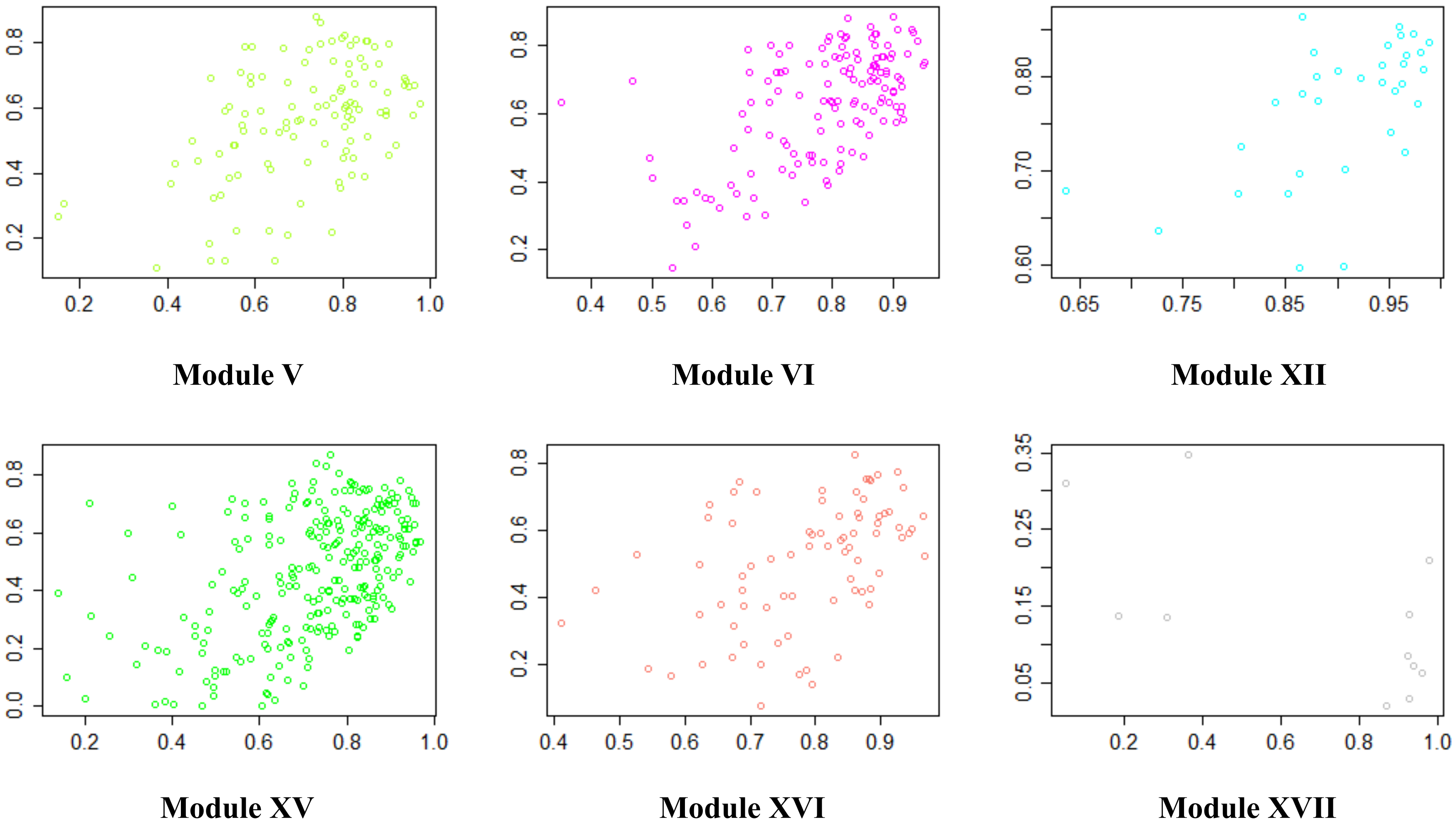

Suppl. Fig. 1 - Correlation between biofuel module membership with biofuel stress response. X-axis indicates the correlation with module eigenpeptide and Y-axis indicates the correlation with biofuel stress-treated phenotypes.
